# QACDes: QoS-aware context-sensitive design of cyber-physical systems

**DOI:** 10.1038/s41598-024-69371-x

**Published:** 2024-08-16

**Authors:** Subhajit Sidhanta, Chanachok Chokwitthaya, Yimin Zhu, Supratik Mukhopadhyay

**Affiliations:** 1 Indian Institute of Technology Kharagpur, Kharagpur, 721302 India; 2 Umea University, 90187 Umea, Sweden; 3 Louisiana State University, Baton Rouge, 70802 USA

**Keywords:** Civil engineering, Electrical and electronic engineering, Sustainability

## Abstract

There is a lot of confusion and ambiguity regarding the quantification of the Quality of Service (QoS) of a system, especially for cyber-physical systems (CPS) involved in automating or controlling the operations in built environments and critical urban infrastructures, such as office buildings, factories, transportation systems, smart cities, etc. In these cases, the QoS, as experienced by human users, depends on the context in which they (i.e., humans) interact with these systems. Traditionally, the QoS of a CPS has been defined in terms of absolute metrics. Such measures are unable to take into account the variations in performance due to contextual factors arising out of different kinds of human interactions. Further, the QoS of a CPS has typically been evaluated by comparing the performance of the actual, fully realized system with the given QoS constraints only after the actual system has been completely developed. In the case of faults in the design exposed by observed deviations from the QoS constraints due to unpredicted variations in the contextual factors, the system needs to be re-designed and re-developed from scratch. Due to the above-mentioned reason, the validation approach associated with the traditional QoS makes the design of CPS systems prohibitively expensive, impractical, as well as infeasible in numerous application areas, such as civil and engineering works, since it may not be possible to modify the system once developed beyond a certain extent. To that end, we propose a context-aware definition of QoS of a CPS which facilitates the design of robust systems as elaborated below. In this paper, we define QoS as a function of contextual factors. A CPS designed according to our QoS specifications would always satisfy the QoS irrespective of any possible changes in contextual factors resulting from many different human interactions that may occur during operation of the system. We also present QACDes - a novel framework that provides a formal mechanism for validating the design of a CPS with respect to the specified QoS constraints at the design phase as well as after the realization of the actual system. QACDes can validate any given CPS, irrespective of its application domain, against a QoS guarantee: (A) as early as even before the design phase by comparing the proposed model with a baseline model, or (B) after the realization of the actual system based on logs collected from running the actual system. We consider a lighting control system that manages the light switches - switching it on/off depending on contextual factors, such as the presence of occupants and time of the day. Using the lighting control system in a building as a use case, we analyze and demonstrate the effectiveness of our QoS definition as well as the QACDes framework against the performance metric measured in an actual fully-realized CPS.

## Introduction

Cyber-physical systems (CPS) have become an integral part of modern built environments and critical urban infrastructures, such as smart cities, smart offices, smart factories, intelligent transportation infrastructures, etc. In such systems, the physical infrastructures are integrated with networked sensors and special-purpose embedded systems such as microcontrollers. The above CPSs automatically actuate the underlying infrastructures to perform various kinds of critical, repetitive tasks, such as controlling the lighting, heating, monitoring traffic, etc. Such CPSs are supposed to conform to safety, sustainability, and energy efficiency constraints, i.e., energy consumption, pollution, redundant actions, unsafe operations, etc., are minimized and simultaneously, the performance objectives of the CPS are satisfied.

**Figure 1:**
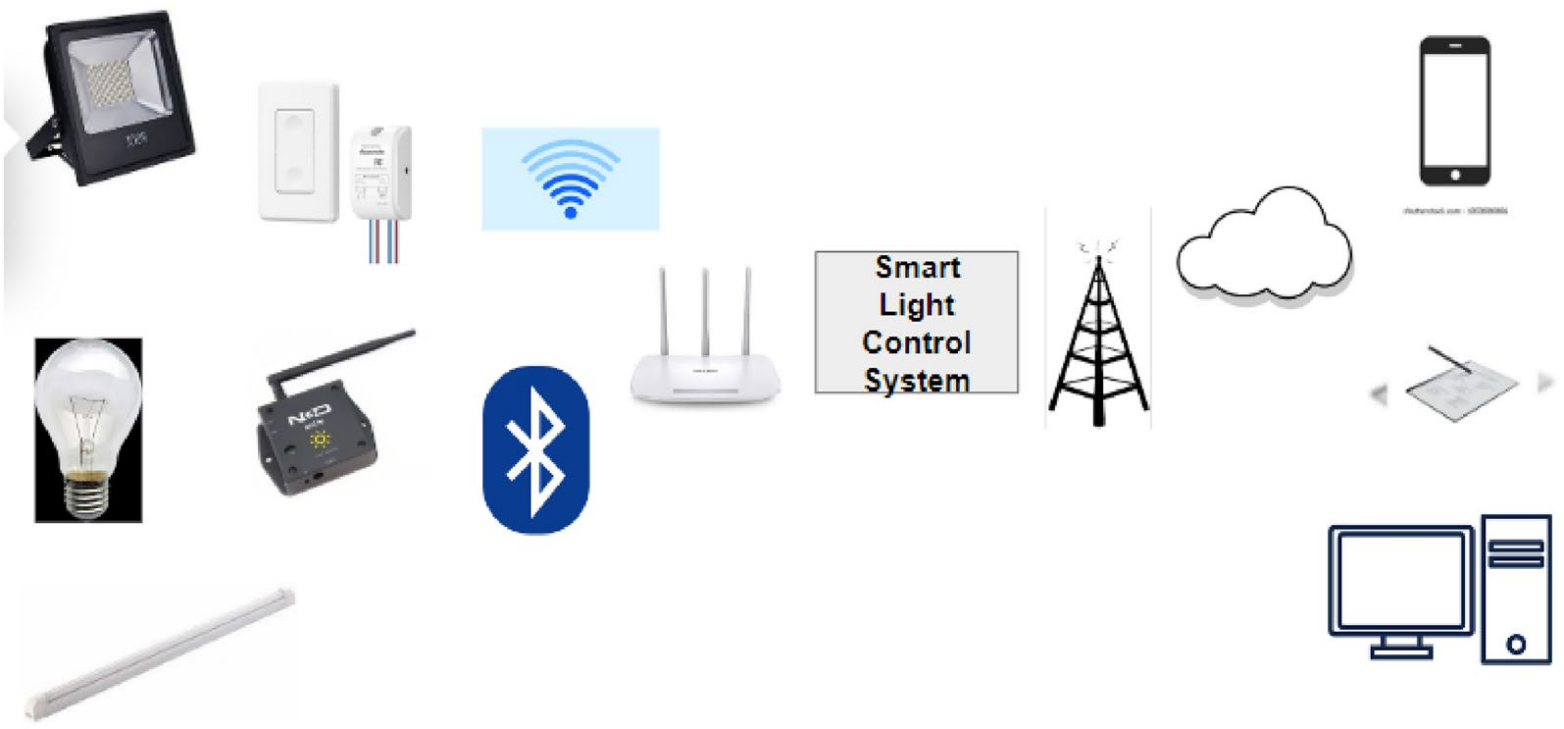
Running example: CPS for lighting control.

A QoS is a constraint imposed on the observed performance of the system, such as an admissible range of values of a chosen performance metric for the performance of the system to be deemed satisfiable. The system either satisfies these guarantees completely or not at all. In this paper, we present the design of a generic framework that can be leveraged to realize a correct CPS design that (A) can satisfy its performance objectives without any need to update the design, and (B) does not require the system to be materialized (i.e., implemented) to test its QoS. Though the proposed approach can be applied to any given built environment, as a running example in this paper, we specifically consider the design of a CPS called a building lighting system, typically deployed in office buildings, which is used to control the lighting in a building (refer to Fig. 1). The QoS of the above CPS is expressed in terms of the minimum illuminance level in a room, given in lux. The primary design objective of the above CPS is to leverage sensors and electronic actuators attached to the electrical switchboards for systematic control of the lighting in a building according to the switching behavior of the users as well as other contextual factors such as occupancy pattern, the artificial lighting status, the lighting intensity, the indoor air temperature, and the relative humidity of the building^1^. Prior literature has already shown that human interactions with the built environment are one of the primary contributory factors to the fluctuations in the energy consumption that occur specifically in office buildings^2^. However, the existing design models of CPS infrastructure and control units do not consider how QoS is affected by human interactions, such as switching behavior, or humans entering and leaving. We argue that a stable design of a CPS for built environment must consider the variability in human interactions to be able to satisfy different performance goals, including minimizing energy consumption during operation.

In most CPS systems such as lighting controllers, traffic monitoring, vehicle routing, etc., the Quality of Service (QoS) guarantees have to be stringently maintained in the presence of dynamic changes in the contextual factors. The above aspect is one of the primary concerns of CPS system developers and service providers while realizing the design of such systems^3–9^. However, QoS guarantees are usually expressed in terms of absolute metrics, i.e., the performance threshold in the QoS for each class of system is defined quantitatively in absolute terms as restrictions on the range of acceptable values of performance metrics. However, it has been established that human interactions with a CPS affects the performance of the system^10^, and thus, in turn, human interaction affects the QoS. It is also recognized that human interaction with a system depends on contextual factors (Context refers to the properties that characterize the nature of the interaction of humans as well as the environment with a system.) that include environmental conditions, age, educational background, familiarity with the system, occupational background, geographical/ethnic background, etc^10^.

Following the above line of reasoning, the QoS of a CPS depends on contextual factors. In reality, QoS should quantify how the *user perceives* the performance of a system in a particular context, i.e., whether the performance of a given CPS observed by the user satisfied the QoS in that context. Ideally, if a system is said to satisfy QoS, it should imply that it does so relative to the context. As per the existing body of research in this area, QoS has not yet been defined in terms of the context in which the system is used^11–14^. There has been a considerably large body of work devoted to the development of making systems context-aware, i.e., able to better adapt to the situation at the time of execution and the execution environment^15–18^. The importance of considering the dynamic nature of the context of interactive applications for designing smart services and systems has been acknowledged by the research community for a long time, especially in the case of CPS and web-based applications which are used by users from diverse backgrounds and regions^19–27^. A section of researchers in the CPS domain has dedicated to the research effort concerning the adaptability of services and personalization of interactions of users with the services through intelligent context modeling and management techniques^25^. There has been a lot of research aimed at improving the following phases in the context management process—context acquisition phase, i.e., a gathering of contextual information, context handling, i.e., deciding whether to adapt the system, and maintenance, i.e., disposing of irrelevant contextual data^26^. A large section of researchers has also worked towards infusing software systems with the ability to adapt security aspects such as access control, and the ability to enable authorization with dynamic context^28,29^. Researchers have also analyzed the trade-off between the complexity arising with modeling of dynamic context and adaptability, i.e., the ability to make accurate predictions even with changing context^27^. Several works have also analyzed the effect of contextual factors on the non-functional aspect of software design to prevent critical bugs in the design^30^. However, to the best of our knowledge, QoS guarantees of cyber-physical systems, in their current form, fail to reflect the true nature of service quality which is affected by changes in the context. Failure to consider the above variations in context gives rise to a discrepancy between the performance of a system predicted during design and the observed runtime performance, which, in turn, can lead to violation of QoS guarantees during operation. Consequently, the state-of-the-art QoS-aware designs of CPS infrastructures may fail to satisfy the QoS at run time with slight changes in the context resulting from variations in human interactions or changes in environmental conditions. With the lack of a robust QoS-aware design, the CPS might need to be redesigned and developed from scratch again, which can be prohibitively expensive, impractical, as well as infeasible in many domains, such as civil and engineering works, since it may not be even possible to modify the system once developed beyond a certain extent.

To that end, we propose leveraging a limited number of IVE (Immersive Virtual Environment) experiments to design a context-aware model of the CPS system. The above model is represented in terms of a special-purpose state machine, where the states denote the conditions of the CPS given by the values of the QoS metrics while the state transitions denote the various interactions of the users with the CPS. The various user interactions with the CPS are quantified in terms of the specific values of the contextual parameters embodied in the labels of the corresponding state transitions. The above design renders our model of a CPS context-aware.

We also present the design of QACDes—a novel framework for validating any given CPS, irrespective of its application domain against a QoS guarantee: (A) as early as even before the design phase by comparing the IVE model with a baseline model, or (B) after the realization of the actual system based on logs collected from running the actual system. To validate if the proposed IVE model is sound with respect to the context-aware QoS, one would typically have to analyze the system logs collected from the IVE experiments to check if the system satisfies the QoS for all possible human interactions. However, collecting the system logs for all possible human interactions is practically impossible given the exponential explosion of test cases for which the IVE experiments need to be run. To the best of our knowledge, QACDes is the first framework that provides context awareness while designing a CPS that satisfies QoS. To use QACDes, the user first has to model the requirements of the system in terms of a special-purpose state machine diagram. Then, IVE experiments are performed on the said state machine, generating an IVE dataset. The above IVE dataset goes through some translations before it is mixed with the existing dataset, which, in turn, is generated by running Monte Carlo simulations on closed form expressions modelling the QoS requirements of CPS systems, such as Hunt’s model. Subsequently, QoS validation is done by comparing the prediction probability obtained from the above-mixed model with those as per a mixture model combining: Model-I) an ideal model of the variations in the performance parameters (output vs input parameters), and Model-II) another model depicting the variations in performance with respect to the variations in the contextual parameters. Following the literature of the concerned class of CPS systems, we choose BPMs (i.e., Building Performance Models) such as Hunt’s model, as the candidate for model-I for our running example. The context-sensitivity of the proposed design quantified by model-II is validated against a performance target, which gives us the distribution functions that quantify the variations of the contextual parameters with respect to the input and output parameters. Da Silva Model^1^ is a logistic model of electric lighting state expressed as a function of the daylight availability and duration of absence (previous and posterior). For the purpose of this paper, the generally accepted Da Silva model serves as our performance target.

As input, QACDes accepts: (A) the QoS guarantees given in terms of the performance metrics, and (B) the ideal performance model, which, in this case, is the BPM. The latter provides the ground truth for validating the design that the QACDes framework proposes. If no such suitable model is there, some requirements may be obtained from various sources, such as use case descriptions, user experience studies, or different standardization agencies. The framework validates the proposed design, expressed in terms of a model generated leveraging IVE experiments, against the inputs provided by the users. Users can also validate the said design of the system with respect to the actual fully realized system. To summarize, this paper makes the following contributions.

- We propose a principled definition of the QoS of a CPS in terms of contextual parameters, which, in turn, is leveraged to devise a context-sensitive design of the system.
- In the absence of a fully realized CPS in the design phase, we use IVE experiments to model the performance of a system under changing contexts through various human interactions.
- Based on the above definition of QoS, we present the design of a framework that can validate the QoS of a CPS in the design phase with respect to a human interaction model of the system developed using IVE experiments.
- Using lighting control systems in buildings as a use case, we analyze and demonstrate the effectiveness of our QoS definition as well as the QACDes framework using data collected from IVE experiments by comparing with the performance statistics collected from an actual fully-realized system.

## Background and motivation

Traditionally, only static aspects of context have been, if at all, considered while designing a system. With reference to a given system, context can be anything from temporal context, which differs according to the time at which the system is observed, to spatial context, which varies with the location and geographical origin/ethnicity of the (people of the) region where the system is being deployed. Further, the common approach followed while designing a context-aware system is to enable the system to adapt to changes in the context at run time. To date, researchers have proposed both off-the-shelf mechanisms and tools to automatically tune the system configuration with respect to the changes in the context. However, such adaptations take time and can be limited by physical/hardware constraints, for instance in civil and engineering domains. Hence, a system would have better chances of satisfying QoS guarantees if the contextual factors are considered right from the design stage, rather than much later during operation. To that end, we present the formal definition of context-aware QoS which can be used to specify the desired QoS of a system at the design time. As a result, a system designed under such QoS constraints always adheres to its QoS despite any dynamic changes in context due to variations in the nature of human interactions with the system.

Unlike the state-of-the-art techniques for context-sensitive design, our proposed approach can validate any given design of a system against all possible variations in the contextual factors using a small sample of data points collected from IVE (Immersive Virtual Environment) experiments. In contrast with modeling-based techniques that use machine learning-based exhaustive training methods to create digital twins of the designed system, we propose a novel approach that allows us to validate the system with limited IVE-based experiments. Normally in machine learning-based techniques, we try to generalize based on exhaustive training against various possible values of the features in a model. In doing so, we do not want the trained model to overfit to a specific set of features which would restrict the model to only a small sample of the population. In our case, we want to induce a bias in our model to enable it to fit a section of the population characterized by a desired set of values for a particular set of contextual factors as per the requirement of the system, e.g., the illuminance thresholds and the occupancy pattern of users for the lighting case study. Further, in machine learning-based techniques we assume that training data is readily available, whereas, in this case, the actual data (that the system needs to be trained on) is available much later when the actual system is fully realized. In our case, we are trying to predict the ideal behavior of the system where the training samples are available much later. Hence, we follow a different approach in approximating the training data using a sample dataset obtained from IVE-based experiments that approximates actual behavior.

Also, the prior techniques do not consider the effect of human interactions in the benchmark experiments intended to validate the QoS of a system; the state-of-the-art papers in the current literature consider user interactions in separate sets of experiments designed to test context awareness of the system. However, the usability of a system is one of the most essential parts of the QoS of a system, and the actual quality of a system makes sense only when it is measured from the perspective of the users of the system. Considering the QoS while designing a system essentially improves certain aspects of human interactions with the system such as ensuring the ease of usage, minimizing human-centric decision-making while operating the system, etc. To that end, we propose to define QoS essentially in terms of a set of user-centric goals, such as improving the energy behavior and the comfort level in a building, ensuring the lack of congestion, timeliness, and safety of commuters in intelligent transportation systems^31^, etc.

## Overview of the approach

In this paper, we consider the use case of an energy-efficient building design considering the light switch behavior of occupants to explain and demonstrate our proposed approach of accounting for the variations in the QoS parameters with respect to changes in various contextual factors during the design phase itself. The performance of such systems (both in terms of user satisfaction and energy efficiency) is dependent on the nature of human interaction occurring with the system, such as switching on/off artificial lights, which in turn, depends on contextual factors such as the presence of occupants or intensity of ambient light. Data about human interaction with a CPS is not readily available at design time, since the system itself does not physically exist at that point. Hence, a QoS-aware design is not practically possible, since the performance of the system cannot be estimated correctly in the absence of the above data. This has prompted us to leverage IVE experiments to generate seed data, which we expand using augmentation techniques, to build the above dataset.

Following a simple approach, the lighting requirement in a room can be estimated by dividing the total light requirement of a room by the light output (lumen) from a single lamp. This simplistic approach for estimating the lighting requirement of a room in an average building is far from practical in a realistic scenario where several other contextual factors, data about which may not be available at design time, need to be considered in the design because the QoS is affected jointly by these contextual factors. For incorporating all possible variations of contextual factors in the design, a vast number of IVE experiments need to be conducted, which may not be feasible in most cases because of the tedious manual work associated with such experiments, as well as the exponentially large possible permutations and combinations for the various parameters. Since there is a lack of availability of data regarding human interaction with a CPS during the design time since the system is not yet realized. Hence, this paper proposes to use a synthetic dataset generated using a limited number of IVE experiments statistically augmented with the Hidden Markov Model (HMM) techniques, to model human interactions with a system. Using the above augmented IVE-based model, we specify the QoS of a CPS in terms of contextual factors, which in turn can be used to realize a context-aware design of a system. Though we have used the example of building design, the same approach can be applied to design problems in other domains as well, such as intelligent transportation systems^31^, where QoS-aware design needs to be performed for constructing context-sensitive systems such as context-aware route recommendation and dynamic navigation systems which are becoming increasingly important in the current age^14^. The proposed technique of system design is also quite relevant for financial services such as stock prediction and financial health analysis, whose accuracy varies radically with changing contextual factors as well as user behaviors (e.g., user’s risk perception that depends on contextual factors)^13^.

## Related work

A section of the research community, especially for large and complex CPS systems^32,33^, has studied properties that affect QoS)^34^, and many researchers have come up with technology and middleware to provide improved QoS awareness in CPS systems^3–9,12,35,36^. Specifically, there has been a considerable effort focused on building adaptive CPS systems that can maintain QoS guarantees while responding to changes in context and environment^11^. There also have been some developments in the estimation of QoS of existing CPS systems, and in techniques to improve the QoS with location-aware personalization techniques^12^. The state-of-the-art techniques for improving the QoS of CPS systems are inherently dependent on the performance statistics collected from a target CPS system, which in turn, renders it dependent on the presence of an actual fully realized CPS system. While QoS has traditionally been the popular criterion for quantifying the performance of systems, there is a general lack of research in the direction of choosing a unified metric for quantifying QoS, and for validating a system against the QoS during the design phase itself. Researchers have had the notion that the QoS metrics pertaining to the network are the most important quality measures for CPS systems^37,38^. However, CPS systems in the current era do not solely imply wireless network systems but a group of systems that can be much larger and more intricate such as lighting and Audio-Video systems for buildings, autonomous driving systems, and streaming applications. Hence, network quality or real-time constraints may not be the most suitable QoS metrics for all CPS systems.

Further, there has been very little work on capturing or modeling human interactions with a system, mostly because of technical as well as ethical challenges in doing so^39^.

Causality has been traditionally chosen as a popular tool to analyze the causal sequence between QoS and QoE of a system^40–43^. Dynamic Causal^44^ modeling is an approach to studying a system as an automaton and analyzing the dynamic cause-effect relationship between states in the said automaton. Dynamic causality has been incorporated in the system models to adapt the model to changing contextual data for maintaining the QoE of the system^42,45^. Dynamic Bayesian Network has been the most popular approach in modeling complex events and systems that have an inherent correlation among the parameters of the model in diverse fields like speech recognition and web search based on contextual information like relevance of clicks^46–48^. Dynamic Bayesian network has been also applied to model complex systems with dynamic context and inherent dynamic causality^49^. A combination of immersive virtual reality and machine learning techniques^50,51^ has been used to improve the accuracy of building performance models. In such applications, to prevent overfitting of machine learning models, synthetic data generation techniques have been used^52^. In^53^, the robustness of such combinations of immersive virtual environments with machine learning models have been investigated. In^54^, causal inference has been used to understand the thermal state and energy behavioral intentions of occupants in buildings. Time series analysis has been used^55^ to validate immersive virtual environments as platforms for collecting contextual data about building occupants.

## QoS definition: choice of QoS metrics

For the design of a lighting system for buildings discussed as an example in this paper, we have opted for a relatively easy-to-measure metric as the output parameter, namely the minimum illuminance level in a room expressed in terms of lux. The contextual factors considered in state-of-the-art building designs are occupancy pattern, the artificial lighting status, the lighting intensity, the indoor air temperature, and the relative humidity of the office^1^. Among the above list of parameters we consider only the occupancy pattern and the lighting status of the office as the main variables because the objective here is to provide systematic control according to the switching behavior of the users; the indoor temperature and the relative humidity were designated as control variables. The occupancy pattern is captured jointly by the variables’ type of leave and the length of the leave for each recorded leave of absence taken by occupants of each room. In this paper, we consider only the occupancy pattern and the illuminance of the office as the model parameters because the objective here is not to maintain the comfort level of users but to provide systematic control according to the switching behavior of the users. We use the suffix *i* in each parameter to denote the *i*th observation in a series of IVE experiments performed on the design of a lighting system to validate the system against the specified QoS. The contextual parameter $Type_i$ denotes whether the nature of the stay is long or short, $Length_i$ is used to denote the duration of a stay, and $occ_ status$ denotes whether a room is occupied or empty. The input parameters $minLux_0$. $minLux_1$, ...., $minLux_n$ quantify the minimum required illuminance, and output parameters $Lux_0$, $Lux_1$, ...., $Lux_n$ denote the observed illuminance at each area in a room. $A_i$ denotes the artificial lighting status, i.e., whether the light in a room is switched on or off.

## QoS specification

To design a context-aware CPS, its QoS must be expressed as a function that restricts output parameters of the system to a certain accepted tolerance threshold, i.e., upper and lower bounds, for all possible combinations of values of the input and contextual parameters (Here we assume that the possible range of variations for the contextual parameters as well as the input parameters are known to us.). The above principle of specifying the QoS in terms of tolerance thresholds which vary with respect to the context of usage ensures that our designed system is able to satisfy QoS in different real-world scenarios characterized by a different range of values of the contextual parameters. For instance, a computer screen display is subject to significantly more stringent performance constraints for a video game application user in contrast to a user who uses the display as an accessory for let’s say proofreading.

For the running example used in this paper, the output parameters are given as different lighting conditions of a room in a given building at different times of the day under different conditions, expressed in terms of the observed illuminance (lux) and light intensity. The input parameters comprise parameters that indicate events such as turning the light switches on/off, occupants leaving the room, and reduction in the external light after sunset. The contextual parameters are the occupancy status, type of stay and length of stay in a given room. In terms of our formal syntax, the QoS guarantee of a system is given as a function of *k* input parameters and the *k* QoS parameters (i.e., the output parameters) $x_1, x_2, x_3,...,x_k$ and $y_1, y_2, y_3,...,y_k$, respectively, where $x_i$ and $y_i$ denote the *i*th input and output parameters, respectively, and output parameters can assume values from the respective domains $D_1, D_2, D_3,...,D_k$ at an instant. Let us consider that the range of the output parameters depends on the value of the input parameters, $x_1, x_2, x_3,...,x_k$ as well as the contextual factors $C_1, C_2, C_3,...,C_l$. For the running example, under a given set of values for the contextual parameters, namely the occupancy status, type of stay and length of stay in a given room, the output parameters, namely observed illuminance (lux) and light intensity, vary directly on the input parameters, namely the boolean parameters quantifying the state of the light switches (on/off), presence of occupants in the room, and reduction in the external light after sunset.

As such, a QoS is given as a function $f_{ qos }({<C_1,C_2,\ldots , C_l>,<x_1,x_2,\ldots , x_l>, <y_1,y_2,\ldots , y_l>})$ which, based on the contextual parameters $C_1, C_2, C_3,...,C_l$, and input parameters $x_1, x_2, x_3,...,x_k$, maps the output parameters $y_1, y_2, y_3,...,y_k$ respectively to sets of permissible values $A_1, A_2, \ldots , A_k$ where for each *i*, $A_i \subseteq D_i$. When the contextual parameters $C_1, C_2, C_3,...,C_l$ are clear, we will denote this function, which we refer to henceforth as the *QoS function*, as $f_{ qos }$. The function $f_ qos$ corresponding to a given QoS can be estimated using estimates $\hat{C_1}, \hat{C_2}, \hat{C_3},...,\hat{C_l}$ of the contextual factors $C_1, C_2, C_3,...,C_l$. If the context changes, i.e, the values of the contextual parameters $C_1, C_2, C_3,..., C_l$ change, then depending on the QoS-function $f_ qos$, the sets of permissible values $A_1, A_2, A_3,...,A_k$ of the QoS parameters also change. Thus, our definition of QoS enforces a system designed under the QoS constraints to be context-sensitive, i.e., adapt to changes in the context.

## Validating a design with respect to QoS

We ensure context awareness in our design methodology for a CPS by allowing for validation of the system against the QoS right at the design phase instead of the traditional way of validating the QoS in a system after it has been actually fully realized (i.e., developed). This ensures that we can fine-tune the design to render the system adaptable to changes in the contextual parameters while preventing violation of a specified QoS guarantee. For the purpose of this paper, we formulate our proposed algorithm and demonstrate it using the use case of the lighting design of a building as the running example. To validate whether a design satisfies the QoS constraint, we have to check the values of the output parameters under a given QoS-function $f_ qos$, with variations in the contextual parameters for different values of the input parameters, against the ideal values as per a baseline model. Hence, our algorithm starts with choosing a baseline model for the lighting system called the BPM model^56^. For the purpose of the lighting control design case study in this paper, we use the widely popular Hunt’s model, given in Eq. (1), as the BPM for our running example, i.e., building lighting system.

Owing to the lack of ability in existing design techniques to accurately model human interaction in a system design, our framework simulates variations in contextual parameters resulting from human actions on the system using IVE experiments. The estimates $\hat{C_1}, \hat{C_2}, \hat{C_3},...,\hat{C_l}$ of the contextual parameters $C_1, C_2, C_3,...,C_l$ can be obtained from appropriately designed IVE based experiments on the system. We use IVE to simulate various possibilities of interaction of the users and the environment with the designed system, and in the process, we validate that the system design satisfies the QoS guarantees as per the QoS design under varying contexts, which in turn, validates the context sensitivity of the proposed design. The baseline values of the QoS parameters obtained from the BPM model are compared with the estimates of the QoS parameters recorded with IVE experiments, elaborated in “Design of IVE experiments”, which emulate different variations in contextual parameters resulting from human interactions with the building lighting system under the proposed design. The context sensitivity of the proposed design is further validated against the Da Silva model.

The traditional approach of validating the soundness of IVE experiments would necessitate running the IVE experiments and comparing the resultant values of the parameters against the QoS guarantee. With this approach, one needs to perform IVE experiments with all possible human interactions and collect the system logs from an exponential explosion of test cases on the IVE setup. In place of this impractical approach, we propose to evaluate the soundness of the IVE experiments in the design phase itself, following the approach presented in^57^ where the IVE experiments are modeled in terms of a state machine representation of the target system denoted as $S_ IVE$. For the running example used in this paper, the states comprised in the state machine $S_ IVE$ correspond to the different lighting conditions of a room in a given building at different times of the day under different conditions, expressed in terms of the observed illuminance (lux) and light intensity. The state transitions in the above state machines represent various interactions of the occupants with the lighting system in the building, such as turning the light switches on/off, occupants leaving the room, and reduction in the external light after sunset. Each of these interactions is quantified by an associated value of the contextual parameters. Similarly, another state machine $S_ Act$ is constructed from the requirements of the lighting system for the concerned building specified by the user or derived from official building design standard guidelines.

A simulation *B* defines a binary relation given as $B \subseteq S \times S$ on the states comprised in a labeled transition system $\langle S,\Delta , \rightarrow \rangle$ such that the following conditions hold for *B*. For every pair of elements $\left( p,q\right) \in B$, $\forall \alpha \in \Delta$, and $\forall p' \in S$, the condition $p \xrightarrow {\alpha } p'$ implies that the condition $\exists q' \in S$ holds such that $q \xrightarrow {\alpha } q'$ and $\left( p',q' \right) \in B$ also holds.

After the above state machines $S_ IVE$ and $S_ Act$ are constructed, we check for the simulation relation from $S_ IVE$ to $S_ Act$, i.e., we check if $S_ IVE$ simulates $S_ Act$. In other words, we verify if each sequence of actions performed in the IVE experiments, given by a state in $S_ IVE$, can simulate a sequence of actions in the real-world, represented by a state in $S_ Act$. This simulation is performed to validate the soundness of the IVE model with respect to that of reality. The simulation check is not performed in the converse direction as the IVE model is not expected to reflect every aspect of reality.

Though the IVE dataset augmented with HMM is able to incorporate all possible variations in the contextual parameters in the design, the design still needs to be validated against some performance model which models the effect of user interactions on the contextual parameters; as mentioned earlier, we use the Da Silva model as the performance target since it quantifies performance as a function of contextual parameters. To that end, after the proposed design passes the pre-validation step (refer to Algorithms 4) performed by the simulation test in the design phase, we apply a generative adversarial network (GAN) to combine the IVE model with an augmented version of Hunt’s model, with the Da Silva model acting as a performance target, such that the IVE model is transformed into a context-sensitive model. Since the Da Silva model is, in fact, a function of contextual parameters, keeping Da Silva as the target of the GAN ensures that the generated model is also context-aware. Once the lighting system has finally been realized/developed from the design, we perform post-validation of the design (refer to Algorithms 2). In the post-validation step, we evaluate how closely the IVE experiments emulate the real-world using the KL Divergence metric which quantifies the distance between the probability distribution functions corresponding to the IVE model and the BPM model, respectively. Algorithm 1 illustrates the detailed steps for validating the design of a system against a specified QoS.

**Figure 2:**
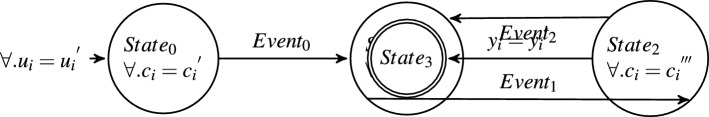
Optimized IVE State model in an intermediate IO-TS form.

**Figure 3:**
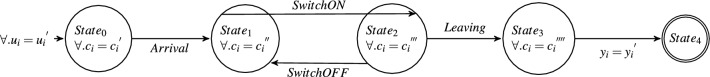
State model for the actual system.

### Design of IVE experiments

Following the experimental setup elaborated by Chokwitthaya et al. in^58^, we design a virtual reality model of a single office of 5.5 $\times$ 4.2 $\times$ 3.2 m, along with estimates for the work area illuminance, using AutoCAD and Autodesk 3ds Max software. Using Unreal Engine 4, IVE experiments on the above were performed by simulating interactions of participants with the above model, such as switching on/off lights and using head-mounted displays projecting the above model. In the training session of the experiments, participants familiarized themselves with the model and the experimental design by exploring the model along with the modalities of the interactions with the said model, mimicking the manner in which they would interact with a light switch as sole occupants in the office which had been modeled. For each event in the experiment session, the model was configured with a different work area illuminance level, and the current conditions of the office were presented to the participants in the form of audio cues. The participants responded to the above contextual parameters by providing the likelihood of switching the light on at that moment. 30 students were used to perform the IVE experiments comprising 48 such events conducted over a duration of 40 min.

### Pre-validation of prospective design

Performance targets can be obtained from benchmark studies in the existing literature or can be based on standards mandated by agencies such as the Department of Energy^59,60^. As per “Residential Lighting: A guide to meeting or exceeding, California’s 2016 Building Energy Efficiency Standards” developed by the California Lighting Technology Center, UC Davis, a building generally has the requirements specified in terms of certain accepted thresholds^61^. Following the above logic, we assume that the recommended threshold for average illuminance is either given by the user in the requirements specifications or specified in building design standards for each type of room in the given building. The threshold for average illuminance, specified as the QoS, is the limiting value against which the illuminance levels (lux) measured in the building are compared. As a general guideline, if the illuminance measured in the building falls below the specified average illuminance threshold even with the artificial light switched on, the current design is deemed unacceptable. A good design must maintain an acceptable illuminance according to the specified lighting uniformity threshold when the lights are switched on.

The state machine representation $S_ IVE '$ of the experiments performed based on the IVE model of the designed lighting system is derived from^57^, where the notation $State\_i$ is used to denote the ’i’th state and $c\_i$ denotes the ’i’th contextual parameter. The state machine in this form, referred to as the Spatial-temporal event-driven (STED) model, is transformed into an equivalent intermediate form called the I/O-TS state machine^62^, defined below. An Input-Output Transition System (I/O-TS) is a variant of the input-output extended finite automata (I/O-EFA) with the omission of internal variables. An I/O-TS is given as a tuple $P=\left( L, U, Y, \Delta , s_e \right)$ where *L* represents the set of states, *U* and *Y* represents the output and’i’^th^ the input, $\Delta$ is the set of all transitions, $s_e$ is the starting input. The above transformation from STED to IO-TS state machine form is performed for the sake of comparing $S_ IVE '$ with the state machine $S_ Act$, derived from the requirements of the actual system illustrated in Fig. 3, given below. $Position_ light ^ sources$ denotes the position of light relative to the windows in each room. The rest of the labels are already described in Section . All contextual parameters in each state $State_i$ are assigned a certain set of values, i.e., $\forall . c_i = c_i'$, where we assign a value $c_i'$ to each contextual parameter $c_i$. After the above transformation, the state machine $S_ IVE '$ is converted to an I/O-TS state machine form, which is further optimized to a compressed version $S_ IVE$ which is illustrated in Fig. 2. The input to $S_ IVE$ is the QoS constraint $f_ qos$, i.e., the minimum acceptable illuminance, and the output is comprised of the contextual parameters plus a boolean variable indicating whether the QoS $f_ qos$ is satisfied. We consider simple electrical switches in all rooms, which can be either in ’ON’ or in ’OFF’ state. Hence the only possible state transitions are switching ON/OFF of lights, and leaving or entering occupants. Contextual factors such as the observed illuminance level (lux) and lighting uniformity determine whether the light switches are turned OFF/ON. Similarly, from the specified requirements of the lighting design of a given building, we construct an I/O-TS state machine $S_ Act$ (refer to Fig. 3) for the actual design of the lighting system in a building.

Let the I/O-TS state machines for the actual design and the IVE experiments be denoted as *A* and *B*, respectively. We check the simulation pre-order between *A* and *B* to validate the soundness of the design. A simulation pre-order relation $SR (A,B)$ is a relation between the two I/O-TS’s *A* and *B* such that: A) every transition of *A* is also present in *B*, i.e., $\forall \delta _e^A \in \Delta ^A. \delta _e^A \in \Delta ^B$, and B) *A* and *B* have identical output, i.e., $Y^A = Y^B$. The details of the simulation algorithm between the state machines $S_ IVE$ and $S_ Act$ is given in Algorithm 4. If the state machine $S_ IVE$ for IVE experiments with a proposed lighting system design passes the simulation check, we mark the proposed design to have satisfied the QoS at the pre-design stage. Otherwise, the framework returns a diagnosis of the above failure in the form of a state machine for the counter-example case.

### Post validation of actual design

We discuss the outline of our approach to the post-validation of the design of a CPS, illustrated in Algorithm 2, using our running example, namely the lighting system of a building. We continue our approach from the last section to use the example of the energy-efficient lighting system design of a building as the use case while discussing our algorithm here. Hunt’s model is taken as the so-called initial BPM with respect to lighting systems. This model expresses the minimum daylight illuminance level in the work area in terms of the lux measured in that area. We denote the minimum daylight illuminance level in the working area *minLuxWorkArea* with the variable *x*, and the term 100*y* denotes the probability of switching on artificial lighting given in terms of a percentage.

1 $$\begin{aligned} y=a+c/(1+\exp (-b(x-m)), \end{aligned}$$

where $$a=0.0175$$, $$b=-4.0835$$, $$c=1.0361$$, $$m=1.8223$$,

$x=\log _{10} (minLuxWorkArea)$. Also, Hunt’s model specifies $y=1.0$ for $x \le 0.843$, and $y=0.0$ for $x >= 2.818$.

In the next step of Algorithm 2, Eq. (1) resulting from the last step is passed as input to the Monte Carlo Simulation algorithm, which runs Monte Carlo simulations on Eq. (1) using a random number generator to generate different values of the input parameter, i.e., $minLuxWorkArea$. The corresponding values of the output parameter, namely the probability of switching the lights ON/OFF, are computed from Eq. (1) for the different values of $minLuxWorkArea$. The above steps are carried out iteratively to produce a dataset for the BPM. Next, we follow the Spatial Temporal Event-driven (STED) modeling approach proposed in^57^ to construct the state machine for the IVE experiments from the requirements specified by the user or given in building regulations documents. A synthetic dataset for the building performance with respect to the lighting parameters is generated by performing IVE experiments based on the above STED model. This synthetic IVE data is augmented by using a hidden Markov model (HMM). In the above HMM, the hidden states and the observation vector corresponding to different events in the building lighting system are assigned as follows. The boolean values denoting the state of the light switch at a given instant are assigned as the hidden states, whereas the corresponding observation vector is formed out of the values of the rest of the parameters, namely occupancy status $os$, intermediate leaving, outdoor illuminance $minLuxOutdoor$, and working area illuminance $minLuxWorkArea$ at each instant. Each observation vector is encoded as an ordinal variable formed by combining the values of each variable comprised in the observation vector described above. For instance, the values of the parameters non-occupancy, short intermediate leaving, bright outdoor illuminance, bright work area illuminance, i.e., values $no$, $short$, $short$, $brightw$, respectively, are combined as an expression $S_ enc : \;mathit{no} + short + brighto + brightw$, which in turn, is encoded as a single value obtained by evaluating the above expression. The transition and observation probabilities of the above HMM are calculated based on the observed values of $S_ enc$ obtained in the observed IVE data. Subsequently, the HMM learns the relationship between the hidden states and observation vectors from the transition and observation probabilities. After completion of the training phase of the HMM, the sequence of events and observations in the IVE data is randomly predicted from the trained HMM model. To implement the above, we have used the python library Emcee which implements a variant of the Markov chain Monte Carlo (MCMC) Ensemble sampling. During the evaluation, this module calls the $run_mcmc$ function of emcee for 500 iterations and returns a Markov chain, which in turn, is used to synthesize the dataset.

Next, we follow in the footsteps of^63^ to apply generative adversarial networks (GANs) for reducing the discrepancy between design-time predictions of system performance, estimated from the IVE experiments, and the observations on the actual system obtained from the existing BPM. As a result, the existing BPM of the design of the lighting system is augmented with knowledge of variations in occupant behaviors in response to contextual factors which is emulated by the IVE experiments. In other words, the use of Generative Adversarial Networks (GANs) has allowed QACDes to generate mixture models that appropriately fuse existing BPM models with knowledge of human interactions obtained from the stated choice experiments in the IVE setup, which results in an augmented model of the system design with enhanced abilities, and ultimately produces designs that provide context-aware QoS. Usually, performance targets and design criteria, specified by an expert designer in the application domain (i.e., construction in our case), are used as guidelines to achieve an appropriate combination of the BPM and the IVE model. In our case, we consider the Da Silva model which is widely recognized as the de-facto standard model for context-aware BPM in lighting systems.

We supply the generator *G* in the GAN^64^ (A GAN is a machine learning framework comprised of two neural networks: a generator and a discriminator.) with two input datasets: the existing BPM dataset *existDataset* generated by the Monte Carlo simulations and the IVE dataset *AugIVEData* produced by the HMM. *G* creates a mixture probability distribution (referred to as an augmented model) by mixing *existDataset* and *AugIVEData* to incorporate within the model the variations in the contextual parameters due to human interactions captured using the IVE experiments. With the above two datasets as input, the GAN performs training to generate an appropriate mixture of the two to satisfy the performance target given by the Da Siva model as follows. A mixture of the datasets *existDataset* and *AugIVEData* is denoted as $\lambda _1 * AugIVEData + \lambda _2 * AugIVEData$, where coefficients $\lambda _1 + \lambda _2 =1$. The GAN is used to estimate the appropriate values of $\lambda _1$ and $\lambda _2$ such that the performance target is achieved. The performance predictions obtained from the augmented model should be as close as possible to the performance targets provided, i.e., the Da Siva model. The discriminator tries to discern the difference between the performance target and the predictions of the augmented model obtained by the generator. The training phase of the GAN involves a minimax game between the generator and the discriminator, during which the generator attempts to learn the augmented model whose predictions satisfy the performance targets^23,65^. Specifically, we have implemented CyclicGAN as follows. The input is passed into the encoder. The encoder extracts features from the input by using Convolutions and compressed the representation of input but increasing the number of channels. The encoder consists of 3 convolution that reduces the representation by 1/4th of actual input size. Then the output of the encoder after activation function is applied is passed into the transformer. The transformer contains 6 or 9 residual blocks based on the size of input. The output of the transformer is then passed into the decoder, which uses 2 -deconvolution block of fraction strides to increase the size of representation to the original size.

We follow Algorithm 3 to validate the system design by comparing the Mixture model generated by the GAN with the model generated by the Da Silva model. For each value of the parameters in the existing BPM dataset, such as illuminance and duration of absence of the user, we estimate the probability *P* of switching ON/OFF of the artificial lights in each room of the building by evaluating the expression in the BPM dataset. *P* is passed on as an argument to Algorithm 3 along with the mixture model. Algorithm 3 computes KL Divergence between *P* and the probability of switching ON/OFF predicted from the mixture model. If the value of KL Divergence falls below the QoS bound specified by the user, we hold the design to satisfy the specified QoS.

**Algorithm 1:**
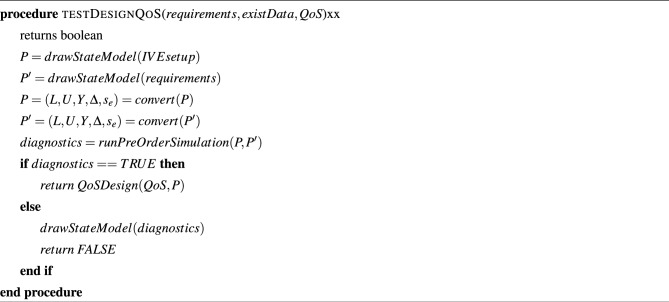
QoS aware design algorithm entry point.

**Algorithm 2:**
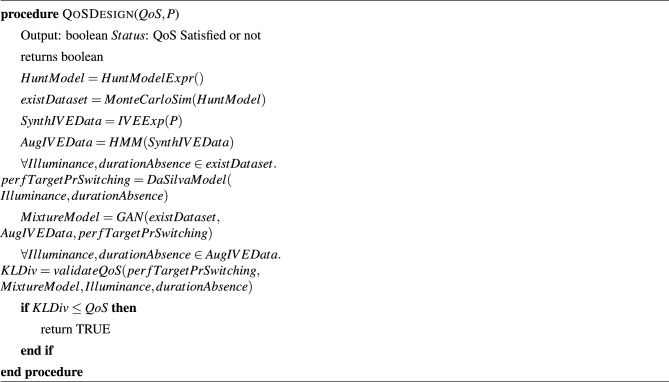
The core algorithm for QoS aware lighting design.

**Algorithm 3:**
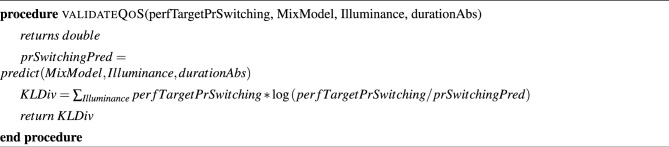
Quantitative evaluation of design.

**Algorithm 4:**
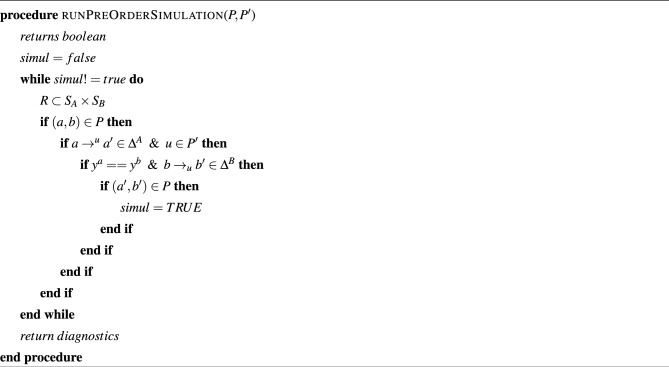
Pre-order simulation for validating design.

## Measuring QoS of a system during design

Hunt’s model expresses the probability *P* of switching light ON/OFF as a function of *illuminance*, whereas Da Silva’s model gives *P* as a function of *illuminance* plus the context parameters, namely $occ_ status$, $Type_i$, and $Length_i$. The probability *P* of switching the artificial lighting ON/OFF is given by the Da Silva model, expressed as a logit distribution function of the parameters, namely *illuminance*, $occ_ status$, $Type_i$, and $Length_i$. The predicted QoS is obtained from the GAN trained on the actual BPM data collected from the commissioned building after it comes into being. In this case, the resultant illuminance is affected by the artificial lighting status, i.e., switching ON/OFF. However, the actual probability of switching ON/OFF does not match the corresponding probability of switching lights in the IVE experiment because of the inherent non-determinism due to the dependency on the contextual parameters. This gives rise to observations where $illuminance < min_ illuminance$, which in turn, results in violation of the given QoS threshold. The QoS is expressed in terms of the percentage of time the condition $illuminance \ge min_ illuminance$ does not hold. This metric is expressed in terms of the KL Divergence, which quantifies the deviation from the expected QoS. QoS derived from historical data about the system can be potentially inaccurate in the absence of information about the context parameters. The GAN is used to generate training data, and it is validated against the real BPM data collected from the augmented version of Hunt’s model or from the commissioned building, combined with the data from the IVE experiments. The new estimated QoS obtained by combining Hunt’s model with the IVE data is validated using KL Divergence.

## Generalizability: intelligent transportation systems

In^31^, Rentala et al. propose techniques to model the causal relationship between traffic crash events and the driver’s behavior with respect to maintaining the stipulated following-distance, i.e., in maintaining a safe distance between the concerned vehicle and any vehicle it may be following. Rentala et al. has leveraged data obtained from IVE experiments to model the impact of the following behavior of drivers on the occurrence (or non-occurrence) of crashes. The experiments as based on a virtual vehicle that is projected on flat 2D screens, integrated with driving simulators to provide realistic driving simulation and modeling of the traffic conditions. Authors have constructed a virtual reality model of the Interstate 10 (I-10) freeway in Baton Rouge, Louisiana to generate IVE experimental dataset comprising participants’ (drivers’) following-distance behavior, from which a forecasting model can be developed to minimize crashes by predicting crashes based on the participants’ following-distance decision. The above model can be improved and validated based on our proposed QACDes framework with a relatively small number of IVE experiments as opposed to the prior work. QACDes can potentially also be applied for different financial applications such as asset pricing, capital market predictions, and financial risk analysis^66^. While ML-based models have been proposed in the literature, there has been a distinct lack of context awareness in the models seen to date. Further, financial data is rarely freely available because of privacy concerns due to which the models trained with scarce data are hardly accurate. QACDes can enable enterprises to build context-aware, accurate models with limited data collected from IVE experiments without any reliance on extensive training data.

**Figure 4:**
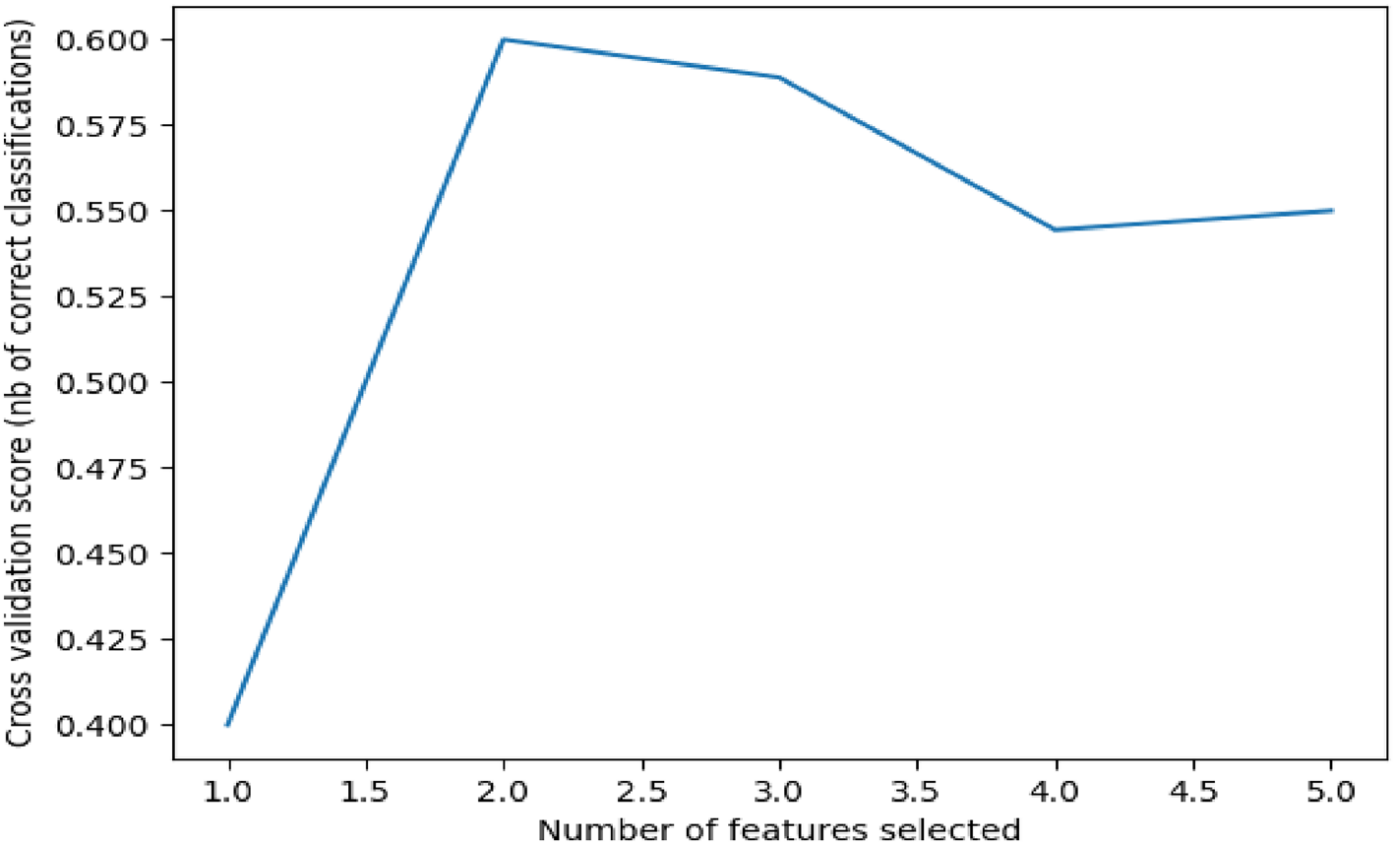
Plot of the number of features VS. cross-validation scores for the HMM step.

**Figure 5:**
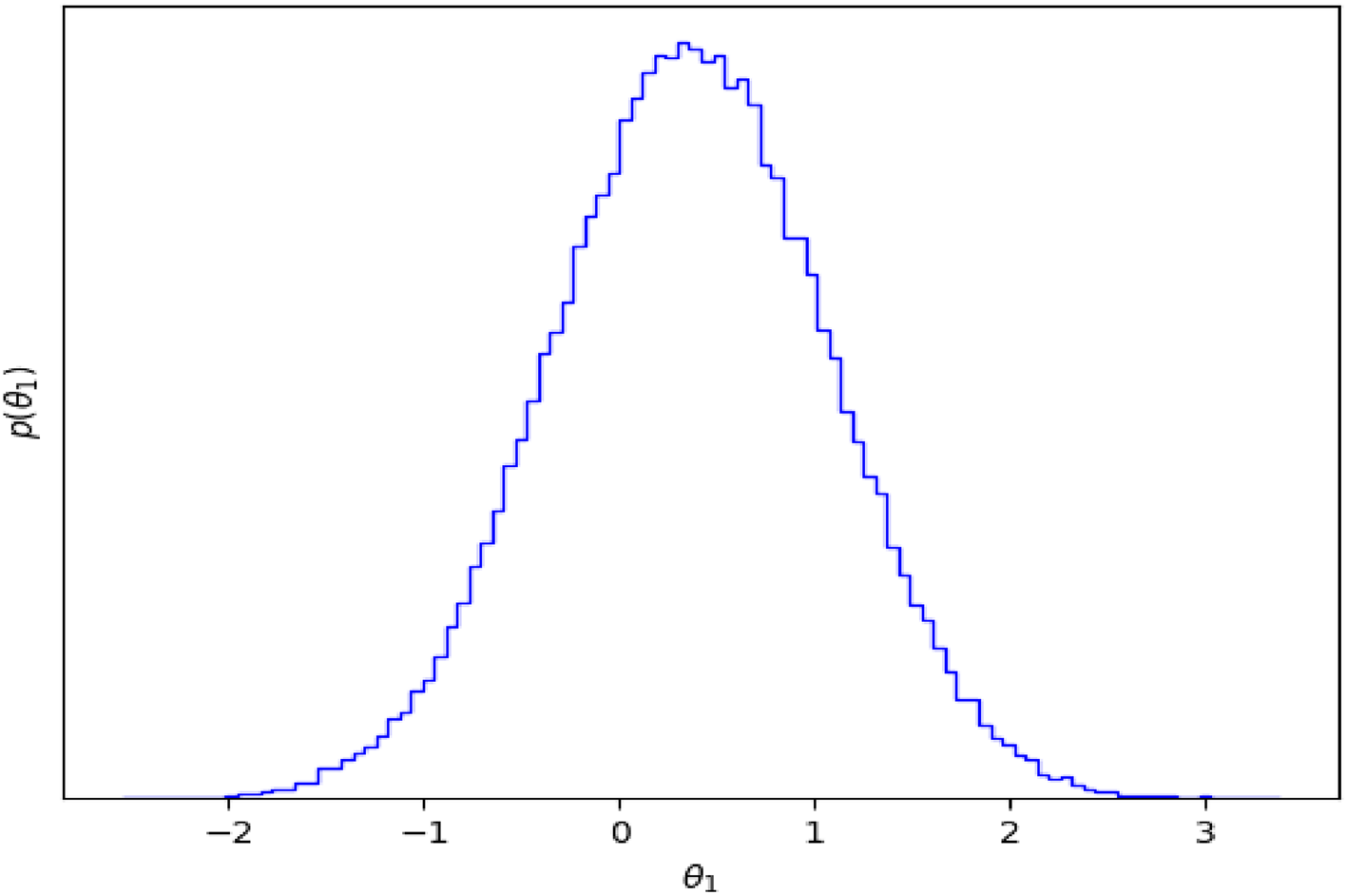
Histograms of MCMC samples to get an estimate of the density that was being sampled.

**Figure 6:**
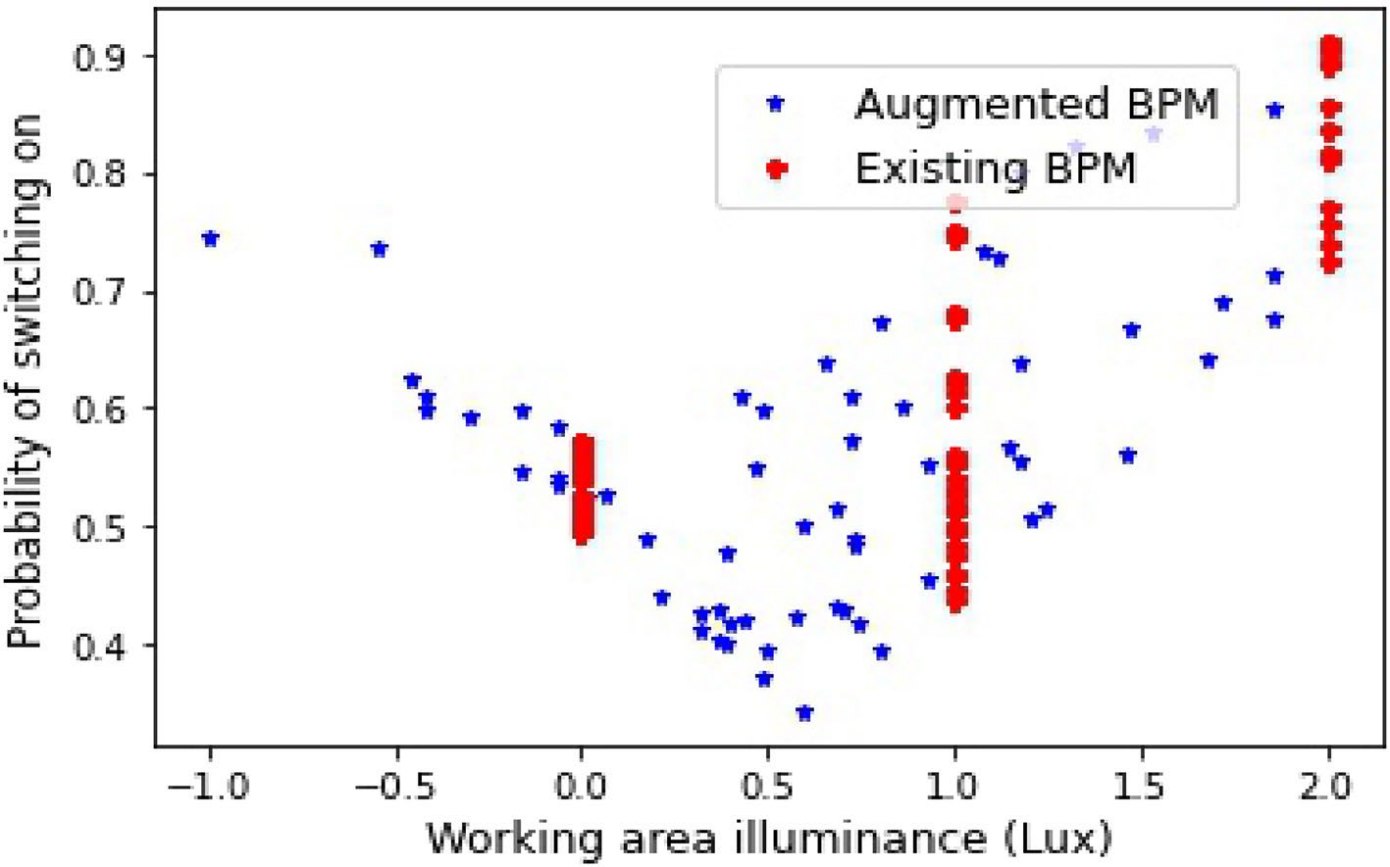
Observations of updated Hunt models obtained from QACDes.

## Evaluation

We demonstrate QACDes by evaluating it on our running example, i.e., the design of the lighting system of a building. As per Algorithm 2, for the purpose of our evaluation, we run the MCMC module of QACDes, used to generate the existing BPM dataset, with 32 walkers where the initial position of each walker is fixed using a random number generation. The MCMC module is run for 1000 steps, generating an array of samples of size 1000 by 32 by 7 where 7 is the dimension of the initial position of the walkers. Here, we choose a multivariate Gaussian probability distribution as the basis for the MCMC sampling. We observe a mean acceptance fraction of 0.552 and a mean auto-correlation time of 57.112 steps for our experiments. Figure 5 illustrates a histogram of the probability distribution of the MCMC samples, which depicts the variations in the density of the samples produced in each step. The simulation pre-order module in QACDes checks for simulation among the state machines for the IVE experiments and the BPM dataset illustrated in Figs. 2 and 3, respectively. The simulation pre-order module gives a success message in case the state machine for the IVE model successfully simulates that for an existing BPM model. Otherwise, QACDes produces diagnostics in the form of counter-examples for the simulation relation. We generated the IVE dataset for the design by running IVE experiments based on the state machine given in Fig. 2. In the HMM module of QACDes, we implemented Algorithm 2 which augments the above IVE dataset using Hidden Markov Model-based Monte Carlo simulation combined with a Support Vector Classification (SVC) comprising a linear kernel. We use cross validation score computed as follows. Cross-validation starts by shuffling the data (to prevent any unintentional ordering errors) and splitting it into k folds. Then k models are fit on (k-1)/k of the data (called the training split) and evaluated on 1/k of the data (called the test split). The results from each evaluation are averaged together for a final score. The HMM module is evaluated using the cross-validation (CV) score obtained for the model trained using the SVC against the total number of features in the dataset, and the resultant CV scores are depicted in Fig. 4. Figure 4 shows a decrease in the CV score as the number of features exceeds 2. With a lesser number of features, the model usually overfits with a training dataset with smaller variation due to the relatively small number of possible values of the small number of features, resulting in a higher cross validation score. With an increased size of feature set, the overfitting is reduced, causing a lower score. Finally, the GAN module QACDes trains a GAN from the above existing BPM dataset combined with the IVE dataset, and this model is further used to predict the probability of switching on lights under a given design. Figure 6 illustrates a comparison between the prediction of the above GAN module with the actual probabilities of switching ON/OFF obtained from the existing BPM dataset obtained by applying MCMC on the existing dataset synthesized from the Da Silva model.

## Conclusions

We have presented an approach of quantifying the QoS of a CPS system with respect to contextual parameters from the perspective of the users of the said system. We have proposed a novel technique to effectively validate the design of the system with respect to the above QoS guarantees, specified by the users or in the design guidelines, in the design phase itself. We presented QACDes—an implementation of our proposed technique that enables validation of the QoS constraints of a system during the design phase itself instead of doing so after the system development, which is increasingly expensive and impractical in general. We have demonstrated that QACDes is effective in validating the design of a lighting system of a building as a use case. In the future, we intend to fix the following shortcomings of the QACDes framework. To be able to produce a good design of a target system, there needs to be a closed form quantifying the dependence of the system performance on the input and contextual parameters, like the BPM for lighting system. Further, QACDes also requires the availability of a target performance model for variations of system performance with contextual parameters, like the Da Silva model, for testing the augmented IVE data.

Future research may branch out from this work by exploring possible application of the framework in other domains, such as intelligent transport system, software engineering, and autonomous driving^67^. Acquiring fine-grained contextual information from satellite imagery^68–70^ should be considered in many of these domains. Use of context-sensitive generative AI^71,72^ for QoS-aware design is another possible area of research, especially aligning^73^ open-source generative AI^74^ to produce systems that respect QoS and QoE constraints. Another possible future direction of research is to explore the possibility of Quality of Experience (QoE)-aware design. Also, the context-awareness of the proposed approach has not been formally validated in this paper; the next task could be formally validating the framework with changes in various contextual parameters in different application domains. QoE has been a relatively newer criterion for evaluating a system. It has already been well established that QoE, which is the human perception of QoS, is, in fact, dependent on the contextual attributes of a system. Despite that, the relation between the QoE and QoS attributes is much more complex, and it remains to be formalized.

We intend to close this gap by developing QACDes into an end-to-end tool that can validate the design of the system with respect to both QoS and QoE even before the actual implementation. The final goal is to develop adaptable and reliable services^75,76^ that respect both QoS and QoE. To the best of our knowledge, our work does not have any ethical implications.

## Data Availability

The datasets used and/or analyzed during the current study available from the corresponding author on reasonable request.
